# The first successful desensitization protocol in exenatide allergy: a case report

**DOI:** 10.1186/s13223-023-00761-y

**Published:** 2023-01-13

**Authors:** Osman Ozan Yeğit, Göktuğ Sarıbeyliler, Pelin Karadağ, Semra Demir, Nurdan Gül, Derya Ünal, Aslı Gelincik Akkor

**Affiliations:** 1 Division of Immunology and Allergic Diseases, Department of Internal Medicine, Istanbul Faculty of Medicine, Istanbul University, Topkapı, Turgut Özal Millet Street, Fatih, 34093 Istanbul, Turkey; 2 Division of Endocrinology and Metabolism, Department of Internal Medicine, Istanbul Faculty of Medicine, Istanbul University, Istanbul, Turkey

**Keywords:** Desensitization, Diabetes, Drug allergy, Exenatide, Obesity

## Abstract

**Background:**

Glucagon-like peptide-1 (GLP-1) receptor agonists are important treatment options in obese patients with type 2 diabetes. To date, few immediate allergic reactions due to GLP-1 receptor agonists were reported. One report revealed that a patient with a level 1 anaphylaxis according to Brighton Criteria due to an exendin based GLP-1 receptor agonist was able to tolerate liraglutide (Human GLP-1 analogue), the alternative GLP-1 receptor agonist. Since exenatide is the only available GLP-1 receptor agonist covered by insurance in Turkey, a drug desensitization protocol, the only therapeutic method in hypersensitivity reactions used in case of absence of an alternative drug, was considered. Here, we report a successful desensitization protocol for the first time in two obese diabetic patients with an immediate hypersensitivity to exenatide.

**Case presentation:**

The first patient was a 47 year-old female. She was referred to our outpatient allergy clinic because of a generalized urticaria developed within minutes after the last dose, following a week of an exenatide BID 5 mcg/20 mcl treatment. Although the reaction was sudden onset, it did not meet the Brighton Criteria of anaphylaxis. The second patient was a 46 year-old female. She had a large local immediate injection site reaction that appeared 15 min following an exenatide BID 5 mcg/20 mcl injection. The injection site reaction was not accompanied by a systemic allergic reaction. We performed desensitization with exenatide to two patients who need GLP-1 receptor agonist treatment. Protocol was completed in 7 steps in approximately 3 h, with the aim of reaching the daily dosage of exenatide. Throughout this process, we observed that both cases tolerated the protocol without any complaints or complications. Following the protocol, the patients safely tolerated the treatment for 3 months.

**Conclusions:**

We present the first successful desensitization protocol to exenatide in both local and/or systemic immediate hypersensitivity reactions and indicate the importance of desensitization in patients who do not have alternative therapies.

## Background

Glucagon-like peptide-1 (GLP-1) receptor agonists, exenatide, lixisenatide, liraglutide, dulaglutide and semaglutide, are among the recent subcutaneous treatment options that provide better glycemic improvement and weight loss in patients with type 2 diabetes according to the current guidelines [1, 2]. Their ability to provide weight loss places them as important therapeutic options for obese type 2 diabetes patients. Exenatide BID is a GLP-1 receptor agonist with proven efficacy and safety which has been in clinical use for over 10 years [3].

To date, few case reports have revealed that GLP-1 analogues can cause allergic reactions [4–6]. One of them showed that a patient with level 1 anaphylaxis according to Brighton Criteria due to exenatide and lixisenatide was able to tolerate liraglutide without any problems [4, 7]. On the other hand, desensitization stands out as a potential option when the use of GLP-1 receptor agonists is required and there is no access to other GLP-1 receptor agonists.

Exenatide and lixisenatide are exendin based GLP-1 receptor agonists. They are structurally similar to exendin-4, a peptide found in the saliva of the Gila monster lizard. In contrast, liraglutide, dulaglutide and semaglutide are structurally analogous to the human GLP-1 molecule [8]. In terms of duration of action, exenatide and lixisenatide are considered short-acting GLP-1 receptor agonists with a half-life of approximately 2–5 h, while dulaglutide, liraglutide and exenatide-LAR are considered long-acting GLP-1 receptor agonists with a half-life of 12 h–several days. Due to its half-life, the recommended dosing interval is twice daily for exenatide [9].

Desensitization is a process that aims to administer essential medications while protecting patients from severe reactions induced by anaphylactic and anaphylactoid mechanisms [10]. Although, desensitization protocols have not been standardized for the treatment of local injection site reactions following subcutaneous medication injections, desensitization can be a valuable choice of treatment in cases of intolerable bothering local reactions [11]. To date, no desensitization protocol with GLP-1 receptor agonists has been reported. Here, we present for the first time two cases, one with a systemic immediate hypersensitivity reaction and the other with a local immediate hypersensitivity reaction due to exenatide, who were both able to receive their drug with the same desensitization protocol.

## Case presentation

### Case 1

A 47 year-old female was referred to our outpatient allergy clinic because of a generalized urticaria developed within minutes in the first week of an exenatide BID 5 mcg/20 mcl treatment. The urticaria developed within minutes after the last dose of injection on the 7th day of treatment, presumably after a 1 week sensitization period. This was the patient's first episode of urticaria. There was no suspected any other allergens such as drug or food exposure as well as the patients did not have any signs or symptoms related to any concomitant infections. Urticaria was not accompanied by respiratory, cardiovascular or gastrointestinal symptoms and disappeared spontaneously after the drug administration had stopped. Although the reaction was of sudden onset, it did not meet the Brighton Criteria of anaphylaxis [7].

**Fig. 1:**
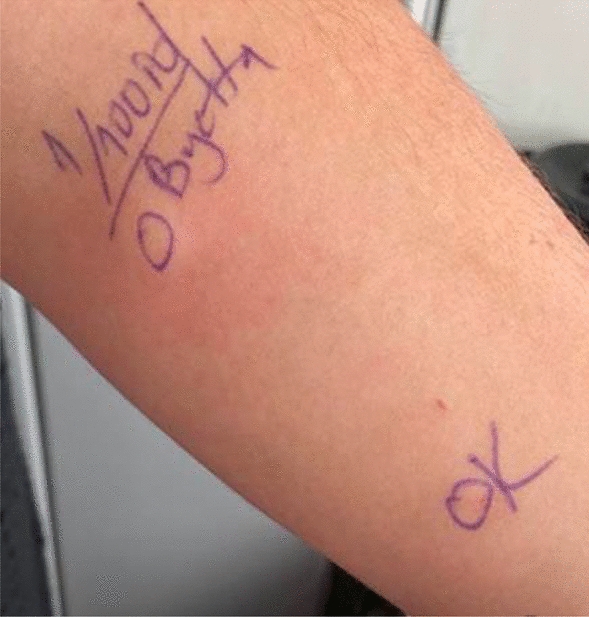
Intradermal test positivity of exenatide in Case 1

**Fig. 2:**
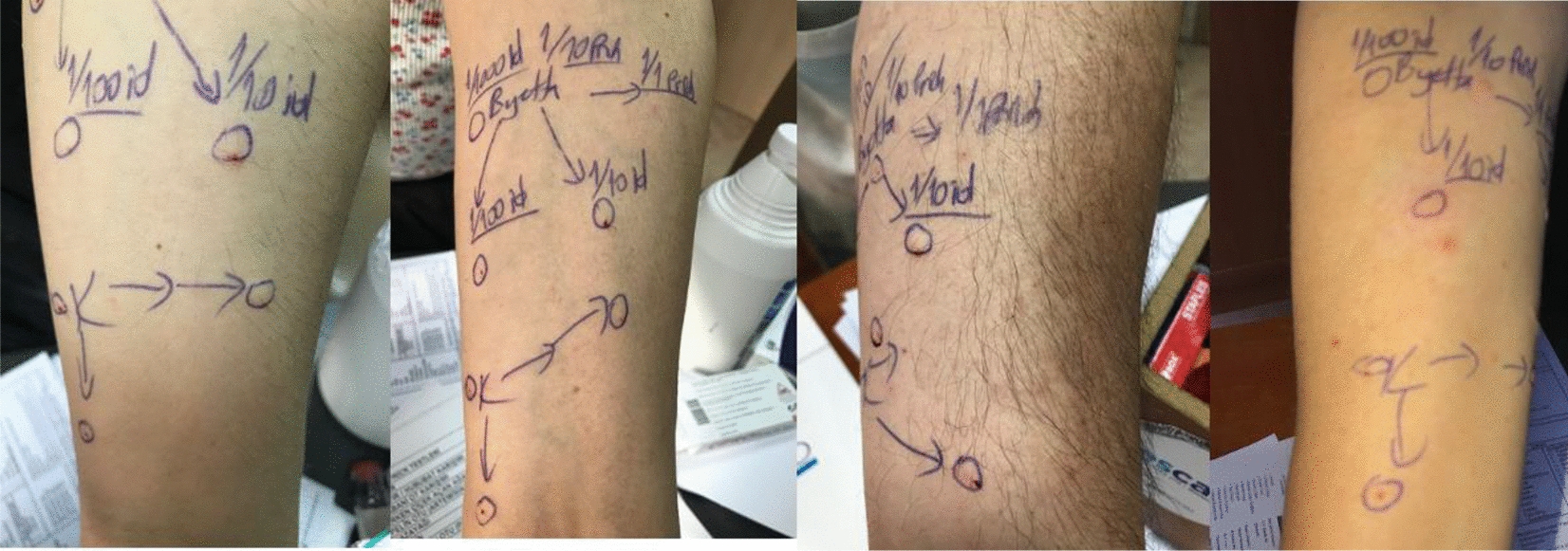
Negative skin test results of the control group

To confirm the diagnosis of exenatide allergy, we performed skin tests with a stepwise approach depending on the previously published report [4]. The skin prick tests were applied with dilutions of 1/10, 1/1 and were followed by the intradermal (ID) tests with 1/1000, 1/100 and 1/10 dilutions respectively until receiving a positive response. The ID test with 1/100 of exenatide was positive in the patient (shown in Fig. 1). Additionally, in order to exclude an irritation reaction that may cause false positivity, skin tests at increasing concentrations were applied to 4 patients who were using exenatide without any problem with the same indication and revealed no positivity (shown in Fig. 2).

We suggested to treat the patient with an alternative treatment. However, it was stated by her endocrinologist that an alternative GLP-1 analogue was not available. Since there was no previously published desensitization protocol with exenatide, we created a new protocol inspired by a previous protocol made for a subcutaneous medication [11]. No premedication was administered before the desensitization. The desensitization process was delivered in 7 consecutive steps of subcutaneous injections with 30 min intervals in our outpatient clinic. The dilution process was carried out with 0.9% NaCl considering the drug content. The targeted daily dose was 10 mcg for the patient. We started with the dose of 0, 1 mcg (1/100 of the targeted dose) than increased the doses with 30 min intervals. In the seventh step, the drug was administered with its own injector and desensitization was completed (Table 1). No allergic reaction developed during the desensitization procedure and the protocol was successfully completed. The patient has been using exenatide treatment for 3 months without any allergic reaction after the desensitization procedure.

**Table 1:** Exenatide desensitization protocol

Step	Time (minutes)	The amount of solution applied in each step (ml)	The dose administered at each step (mcg)	Cumulative dose (mcg)
1	0th	0.04	0.1	0.1
2	30th	0.08	0.2	0.3
3	60th	0.16	0.4	0.7
4	90th	0.32	0.8	1.5
5	120th	0.64	1.6	3.1
6	150th	0.76	1.9	5.0
7	180th	Application with autoinjector	5.0	10.0

Each application was performed with an interval of 30 min and the total dose was determined as the daily therapeutic dose for the particular patient. In the first six steps, 5 mcg/2 ml dose of solution obtained by adding 1.98 ml of 0.9% NaCl to 5 mcg/0.02 ml dose of drug was used. In the seventh step, the drug was administered with its own injector

*ml* milliliter, *mcg* microgram, *NaCl* sodium chloride

### Case 2

**Fig. 3:**
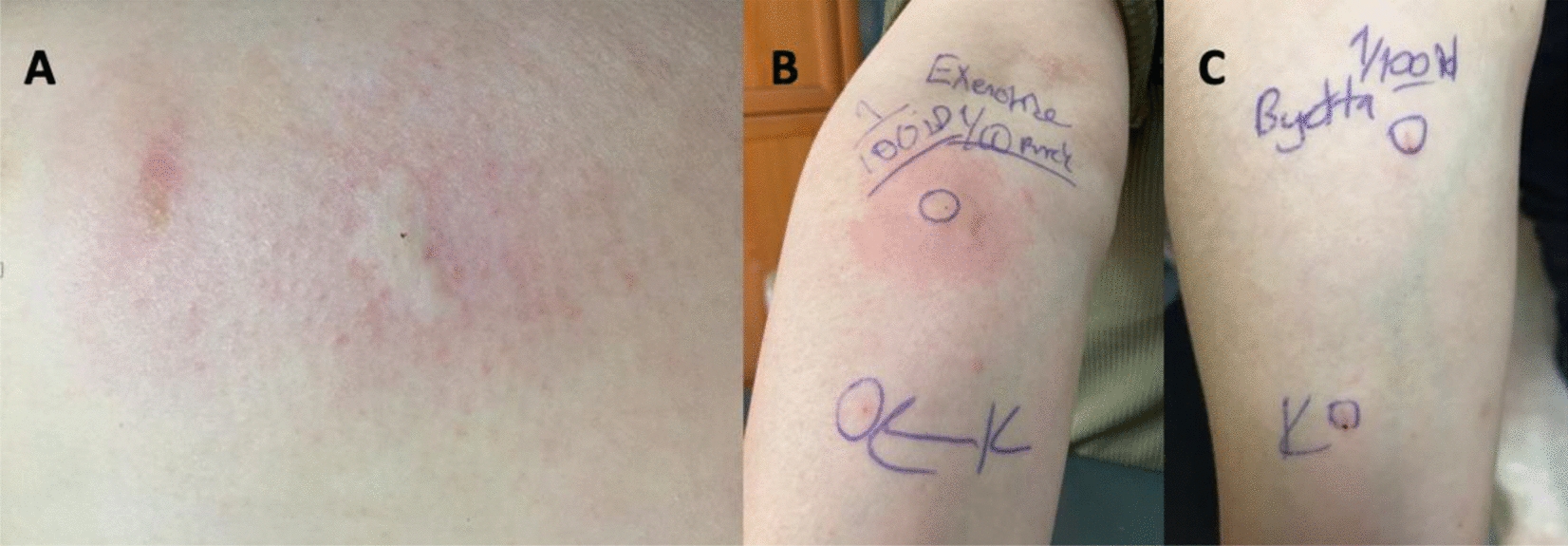
**A**. Local site reaction on the upper posterolateral part of the right arm after exenatide injection in case 2. **B**. Intradermal test positivity with 1/100 dilution of exenatide in case 2. **C**. The negative skin test after desensitization in case 2

A 46 year-old female, diagnosed with type 2 diabetes and obesity, was referred to us because of having a large local immediate injection site reaction appeared in 15 min following an exenatide BID 5 mcg/20 mcl injection for a month. After the fourth injection, an itchy, edematous and hyperemic lesion had developed on the injection site (shown in Fig. 3A). Skin prick and ID tests were applied on the patient with the same protocol and ID test with 1/100 dilution revealed positivity to exenatide (shown in Fig. 3B).

Since the patient did not have a systemic reaction, a divided dose and local steroid therapy was initially administered. However, bothering injection site local reaction continued to occur after divided doses and local steroid usage. Therefore, we decided to apply the same desensitization protocol on the patient who could not tolerate the splitted doses of the drug during long-term therapy. During the protocol, no lesion occurred on the injection area and the patient continued to use the drug in one injection per application in the following days without any reaction. Furthermore, the following skin tests performed in the first month of desensitization were interestingly found negative and the patient has been using exenatide treatment for 3 months without any allergic reaction (shown in Fig. 3C).

## Discussion and conclusions

Drug desensitization is the sole treatment choice in the patients allergic to a drug which has no alternatives. During desensitization, drug antigens are reintroduced in an incremental fashion, allowing to reach full therapeutic doses of the culprit drug with minor or no reaction. Temporary tolerance is achieved in hours and can be maintained if drug antigens are administered at regular intervals, depending on the drug’s pharmacokinetic parameters [10]. Here we report a successful desensitization protocol for the first time in two obese diabetic patients with an immediate hypersensitivity to exenatide.

When an allergic reaction develops due to an exendin based GLP-1 receptor agonist (exenatide, lixisenatide), the usage of a human GLP-1 analogue (liraglutide, dulaglutide, semaglutide) may provide a solution since no cross-reactivity has been reported so far [4, 8]. However, desensitization can be preferred if alternative GLP-1 receptor analogues are not available. Since exenatide is the only GLP-1 receptor agonist covered by insurance payment in Turkey, we had to perform desensitization in these two patients who were proved to be allergic to exenatide.

We assumed that both reactions were immediate IgE-mediated hypersensitivity reactions based on the timing of the reaction and the test results. The test results of the case report of Shamirz O. et al. and the results of our control group showed no false positivity [4]. However, a false positive skin test result is possible given the limitations of skin testing with subcutaneous agents.

Although we could not find any data in the literature, mast cell activation with a non-IgE-mediated way, for example MRGPRX2, is also possible. Besides, there is also the possibility of the allergic reactions may occur not with the active drug, but because of metacresol, an antimicrobial additive. However, both patients were able to use insulin glargine containing metacresol after exenatide allergy.

Our desensitization protocol was completed in 7 steps in approximately 3 h, with the aim of reaching the daily therapeutic dosage of exenatide. Throughout this process, we observed that the patients tolerated the protocol without any complaints or complications during injections. Besides, the daily regular administration of exenatide without dose interruptions enabled us to achieve success in suppressing the immediate hypersensitivity reaction with a single desensitization period. 3 months after the desensitization protocol, our patients were still applying exenatide injections without any allergic reactions. Besides, the skin tests with exenatide of case 2 unexpectedly turned to negative after desensitization. This finding suggested us the possibility of cessation of a local IgE-mediated response to the drug after the desensitization protocol which is uncommon in most of the desensitization procedures.

Temporary toleration is achieved in hours and can be maintained if drug antigens are administered at regular intervals, depending on pharmacokinetic parameters [10]. If the intervals exceed twice the drug half-life, it is recommended to reapply the desensitization protocol. In our patients, re-desensitization was not needed, despite the applications performed in every 12 h. Exenatide has a half-life of approximately 2–5 h but in our cases, it might be prolonged due to its pharmacokinetic parameters. It has been shown that the drug elimination half-life may be prolonged, depending on the severity of obesity and drug properties [12]. Therefore, we can speculate that obesity and increased subcutaneous adipose tissue in both cases may be associated with increased drug half-life and prolonged toleration period.

As a limitation, the fact that both patients successfully desensitized with exenatide had non-anaphylactic immediate allergic reactions, leading to insufficient evidence for the application of this protocol in anaphylaxis. However, we think that the use of this protocol in non-anaphylactic immediate reactions in the presence of appropriate indications will provide the expected benefit.

In conclusion, this first report demonstrated a successful desensitization protocol to exenatide in patients with isolated immediate skin reaction and local reactions and indicated the importance of desensitization in patients who do not have alternative therapies.

## Data Availability

Medical records containing clinical data of patients are available.

## Abbreviations

GLP-1: Glucagon-like peptide-1
ID: Intradermal
